# Prostatic fascia and recovery of sexual function after radical prostatectomy: Is it a “Veil of Aphrodite” or “Veil of mystery”!

**DOI:** 10.4103/0970-1591.45558

**Published:** 2009

**Authors:** Anil Mandhani

**Affiliations:** Department of Urology. Sanjay Gandhi Post Graduate Institute of Medical Sciences, Lucknow, Uttar Pradesh, India

**Keywords:** Neurovascular bundle, radical prostatectomy, veil of Aphrodite

## Abstract

Sexual dysfunction is one of the most controversial aspects associated with radical prostatectomy. Since Walsh's description of neurovascular bundle there have been number of articles describing various modification to the technique of bilateral nerve sparing to augment the recovery of sexual function. There is a very thin line between performing an ideal nerve sparing and giving equally good oncological outcome in terms of negative surgical margin. “Veil of Aphrodite” nerve sparing technique was conceptualized by Menon *et al.* Lately other related terms have emerged in the literature e.g., “high anterior release, “curtain dissection,” or “incremental nerve sparing. Does veil technique of radical prostatectomy help improve recovery of sexual function? Do mere presence of nerves in veil account for potency? Are these nerve parasympathetic? This short review tries to find the answer of these questions in contemporary world literature.

## WHAT IS VEIL AND HOW DOES IT DIFFER IN VARIOUS DESCRIPTIONS!

**Figure 1:**
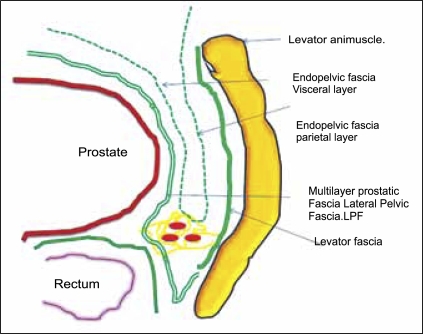
Line diagram showing fascias around the prostate

Endopelvic fascia has the parietal and visceral layer, which covers the pelvic diaphragm and prostate [Figure 1]. Underneath this the prostate is covered with the prostatic fascia anteriorly and anterolaterally. The major tributaries of Santorini's plexus travel within this fascia. Laterally the prostatic fascia fuses with the levator fascia, which covers the pelvic musculature, to form the lateral pelvic fascia (LPF) [Figure 1].[1] In an effort to avoid injury to the dorsal vein of the penis and Santorini's plexus during radical perineal prostatectomy, the lateral and anterior pelvic fasciae are reflected off the prostate, which accounts for the reduced blood loss associated with radical perineal prostatectomy.[1,2]

In Myers's description of fascial anatomy, displaced medial part of levator fascia comprising the most superficial layer and the deeper layers continue to cover the prostate which is akin to LPF.[3]

Neurovascular bundle is outside the prostate between layers of the levator fascia and prostatic fascia. Walsh's description of high release of the levator fascia is that the levator fascia over the anterior apex to the prostate is incised along the lateral edge of the dorsal vein complex, preserving the underlying prostatic fascia. Accordingly if nerve sparing is performed correctly the prostatic fascia must remain on the prostate.[2] In contrast to this the concept of veil is to incise medially at the prostatic fascia or LPF and not to leave even a shred of tissue over the prostate. The correct plane of veil is between the LPF and the glistening surface of the prostate. Once this avascular plane is entered, the neurovascular bundle can be teased away from the prostate easily. The resulting neurovascular bundle is embraced in a veil of tissue, the so-called “veil of Aphrodite.[4]

Another controversy which has risen due to veil technique is that as there is no true capsule in the prostate to diagnose pT3 (extra prostatic extension). Invasion of LPF or dorso-lateral fatty tissue of the prostatic gland is taken as pT3. So with veil technique when there is no LPF left on the prostate then interpretation of the stage would be different.[5]

While the classic description of neurovascular bundles involves 2 well-defined structures lying in a groove between the prostate and rectum, recent studies suggest that accessory neural channels exist in the prostatic fascia that may supplement neural stimulation to the penis.[1] It has been shown that 52% of Radical Prostatectomy (RP) specimens, the nerves were found along the entire lateral aspect of the prostate without any particular location.[1] Theoretically nerves should be present in all the body tissues and mere presence should not support the hypothesis that these are responsible for erection. Moreover nature of these fibres have been shown to be of sympathetic as hypogastric nerve fibres predominate at a more ventral location which may not affect the potency.[6]

In 2004, Costello *et al.* showed that most of the bundle descends posterior to the seminal vesicle. The nerves converge to the mid-prostatic level but diverge once again as they approach the prostatic apex. The anterior and posterior nerves of NVB are separated by about 3 cm at the level of the base of the prostate.[7]

There are contradictory reports of clinical trial for and against preserving veil to be of any help to improve potency. Proponent for doing veil technique reported the first clinical results in highly selective cases. In that uncontrolled study of 154 patients with clinical stage of T1c and mean PSA of 5.1 ng%; 96% of the men were reported to have intercourse. Surgical margin was positive in 5%.[4] In absence of any true anatomical capsule there is always a concern of entering into the prostate while doing veil technique. Though this is the lowest reported margin positivity with veil technique in radical prostatectomy with any approach, we should realize that in this study 99.4% patients had organ confined T1c disease so why should even 5% of these patients have margin positivity?

The same group recently published their results in 1142 out of total 2652 case done. With veil of Aphrodite, return of the base line function was 73% in those who had normal erectile function [based on sexual health inventory for men (SHIM) of more than 21] in comparison to those 39% only with standard nerve sparing surgery. This difference could be confounded with study design which was unmatched, uncontrolled, retrospective in nature with capture rate of follow up results of just 43%. Non responders to any survey on follow up would mean that they have a lower quality of life scores.[8] Even in the select group of 1142 patients, margin positivity was not compared amongst groups who had standard vs. veil of Aphrodite RP.[9] There is only one retrospective comparative study of unmatched groups of 137 patients each, operated with veil and standard nerve sparing radical prostatectomy. Recovery of preoperative sexual function was not different in both the groups and was 68.4% and 67.2%.[10] This contradicts the fact the nerves present in the LPF are responsible for the erection.

## CONCLUSIONS

Ideally any approach to radical prostatectomy should restore the sexual function equal to what patients had before the surgery. Lack of well-designed trial on recovery of function denotes that how little we understand about pathophysiology of erectile dysfunction in relation to radical prostatectomy. Individual interpretation and reporting higher results in recovery of sexual function is in the line with attracting patients to practice and maintaining one-upmanship too. As radical prostatectomy is the most common surgery performed in urological malignancy it would be better to answer these questions by conducting prospective case control trial combined with anatomical studies.
